# Melatonin Attenuates Sepsis-Induced Small-Intestine Injury by Upregulating SIRT3-Mediated Oxidative-Stress Inhibition, Mitochondrial Protection, and Autophagy Induction

**DOI:** 10.3389/fimmu.2021.625627

**Published:** 2021-03-12

**Authors:** Siqi Xu, Lulan Li, Jie Wu, Sheng An, Haihong Fang, Yunyang Han, Qiaobing Huang, Zhongqing Chen, Zhenhua Zeng

**Affiliations:** ^1^ Department of Pathology, Qingdao Municipal Hospital (Group), Qingdao, China; ^2^ Guangdong Provincial Key Laboratory of Shock and Microcirculation, School of Basic Medical Sciences, Southern Medical University, Guangzhou, China; ^3^ Department of Critical Care Medicine, Nanfang Hospital, Southern Medical University, Guangzhou, China; ^4^ Department of Anesthesiology, Nanfang Hospital, Southern Medical University, Guangzhou, China

**Keywords:** melatonin, sepsis, small intestine, SIRT3, mitochondria, autophagy

## Abstract

Melatonin reportedly alleviates sepsis-induced multi-organ injury by inducing autophagy and activating class III deacetylase Sirtuin family members (SIRT1–7). However, whether melatonin attenuates small-intestine injury along with the precise underlying mechanism remain to be elucidated. To investigate this, we employed cecal ligation and puncture (CLP)- or endotoxemia-induced sepsis mouse models and confirmed that melatonin treatment significantly prolonged the survival time of mice and ameliorated multiple-organ injury (lung/liver/kidney/small intestine) following sepsis. Melatonin partially protected the intestinal barrier function and restored SIRT1 and SIRT3 activity/protein expression in the small intestine. Mechanistically, melatonin treatment enhanced NF-κB deacetylation and subsequently reduced the inflammatory response and decreased the TNF-α, IL-6, and IL-10 serum levels; these effects were abolished by SIRT1 inhibition with the selective blocker, Ex527. Correspondingly, melatonin treatment triggered SOD2 deacetylation and increased SOD2 activity and subsequently reduced oxidative stress; this amelioration of oxidative stress by melatonin was blocked by the SIRT3-selective inhibitor, 3-TYP, and was independent of SIRT1. We confirmed this mechanistic effect in a CLP-induced sepsis model of intestinal SIRT3 conditional-knockout mice, and found that melatonin preserved mitochondrial function and induced autophagy of small-intestine epithelial cells; these effects were dependent on SIRT3 activation. This study has shown, to the best of our knowledge, for the first time that melatonin alleviates sepsis-induced small-intestine injury, at least partially, by upregulating SIRT3-mediated oxidative-stress inhibition, mitochondrial-function protection, and autophagy induction.

## Highlights

- Melatonin alleviates sepsis-induced small-intestine injury.
- Melatonin produces this effect partially by upregulating SIRT3-mediated oxidative-stress inhibition, mitochondrial-function protection, and autophagy induction.
- The research may provide further mechanistic support for melatonin for the treatment of clinical sepsis.

## Introduction

Sepsis is a syndrome of physiologic, pathologic, and biochemical abnormalities induced by infection, and severe sepsis can lead to multiple-organ dysfunction syndrome (MODS) (1).To date, inflammation-induced cytokine activation and reactive oxygen formation are now considered to be of central pathogenic importance in sepsis (2); moreover, autophagy inhibition accelerates multiple-organ injury in murine sepsis models (3). Thus, increased inflammatory cytokine and oxidative stress generation and diminished autophagy are implicated in the pathogenesis leading to multiple-organ damage and dysfunction during sepsis. The gut may serve as the motor of MODS, probably because of the disruption of intestinal barrier and the subsequent translocation of intestinal bacteria. The integrity of structure and function of intestinal epithelial cells is crucial in intestinal barrier. The abnormalities of intestinal epithelial cells have been frequently observed in septic patients, which was thought playing a central role in the development of bacterial translocation and systemic infections (4).

Melatonin, a tryptophan-derived substituted indoleamine (**SFigure 1**) widely found in evolutionarily distant organisms (5), is considered as a neurohormone with an impact on circadian rhythm regulation, seasonal reproductive cycles, and mammalian immune system modulation. In terms of combating sepsis, melatonin was initially recognized as a potent antioxidant (6), and it reduces lipopolysaccharide (LPS)-induced oxidative damage *in vivo *(7) and lipid peroxidation *in vitro *(8), probably by inducing antioxidant-enzyme expression, resulting in anti-inflammatory and mitochondrial protection effects(15). The exogenous application of melatonin improves the survival of LPS-induced sepsis in mice (9). Moreover, it can upregulate autophagy during sepsis-induced cardiac dysfunction (10). However, its precise protective mechanism in sepsis is incompletely understood.

One of potential mechanisms for the antioxidant role of melatonin is its effects on Sirtuins (SIRTs) upregulation (11). SIRTs are class III histone deacetylases sorted into groups I–IV (comprising, respectively, SIRT1–3, SIRT4, SIRT5, and SIRT6/7) (12). SIRTs regulate various physiological functions, from energy metabolism to stress response and they also demonstrate important antioxidant activity, mainly for its effects of deacetylation and activation of serious antioxidant enzymes. It has been revealed that melatonin treatment elevates the expression and activity of SIRT1 in various animal/cell sepsis models, and alleviates sepsis-induced brain and liver injury, as well as cardiac dysfunction (10, 13–16). Moreover, melatonin can activate SIRT3 to reduce oxidative stress (17) and inflammation (18), induce autophagy (18), and preserve mitochondrial function (19, 20). Its effect on sepsis is also reportedly related to the regulation of other SIRTs, including SIRT2 (21, 22), SIRT4 (23), and SIRT6 (24).

It has been reported that oxidative stress is an important cause of intestinal barrier dysfunction in the development of intestinal hyper-permeability and bacterial translocation (25–27). However, the effect of melatonin on the intestinal barrier function is rarely reported. This study intended to explore whether melatonin has a protective effect on the intestinal barrier function, and whether its effect is related to its up-regulation of SIRTs and then its antioxidant effect.

## Materials and Methods

### Reagents

SIRT3 inhibitor 3-(1H-1,2,3-triazol-4-yl) pyridine (3-TYP) was synthesized and characterized by the School of Pharmaceutical Sciences, Southern Medical University, Guangzhou, China, based on our previous work (28). Information on other reagents is presented in the Supporting Information.

### CLP-/Endotoxemia-Induced Sepsis Models

This study was conducted in strict accordance with the recommendations in the *Guide for the Care and Use of Laboratory Animals* (US National Institutes of Health, Bethesda, MD, USA), and all appropriate measures were taken to minimize the suffering of experimental animals. The study protocol was approved by the Committee on Ethics in Animal Experiments of Southern Medical University. And all the study was carried out in the Guangdong Provincial Key Laboratory of Shock and Microcirculation in Southern Medical University. Two sepsis models were developed as described before (29, 30). The detailed information is presented in the Supporting Information. All mice were anesthetized through isoflurane inhalation (RWD Lifescience) during the procedure and were sacrificed by cervical dislocation at different timepoints as necessary for blood-sample and tissue extraction. The tissue samples from the intestine were collected and prepared for the determination of oxidative stress state and the expression and activities of SIRT3 and SOD2, etc.

### Tissue-Specimen Preparation and Histopathological Analysis

Lung, liver, kidney, and small-intestine specimens from all animals were fixed in 4% paraformaldehyde, embedded in paraffin, sectioned at 4–6 μm thickness, and stained with hematoxylin and eosin for light microscopy. Quantitative scoring standards were established based on histopathological scores of the lung, kidney, and liver (31). Mucosal damage in the small intestine was assessed using reported criteria (32). Briefly, to ascertain the degree of intestinal damage, small-intestine specimens were oriented along the crypt-to-villus axis during embedding, and injury was graded from 0 to 5; 0: mucosa with normal villi; 1: development of subepithelial space at the villous apex, frequently associated with capillary congestion; 2: scattered epithelial denudation on villous tips; 3: denuded tips with exposed lamina propria and villous blunting; 4: epithelial shedding from both the apex and mid-region of the villi associated with shortened and widened villous structure, as well as exposure of dilated capillaries; 5: complete destruction of villi and disintegration of the lamina propria with ulceration (32). Multiple-organ injury extent was evaluated microscopically by two senior technicians blinded to the treatment protocol. In each tissue sample, 10 random fields were scored, and the mean value was calculated for statistical analysis.

Plasma melatonin and serum cytokines were measured using ELISA. Detailed information is presented in the Supporting Information.

### Organ Function and Injury Assessment

Blood was obtained through retro-orbital bleeding at different time points and was immediately centrifuged (7500 × *g*, 2 min) to obtain plasma; 80 μL samples were used for measuring the biochemical parameters of liver and kidney function: liver-function parameters: plasma levels of aspartate aminotransferase (AST), alanine aminotransferase (ALT), and total bilirubin (Tbil); renal-function parameters: plasma levels of blood urea nitrogen (BUN) and creatinine (CREA). All biochemical parameters were analyzed using a Fuji DRI-CHEM 3030 (Fuji Photo Film Co., Ltd.) (33).

### SOD2 Activity Determination

SOD2 activity was measured using a commercially available kit, with water-soluble tetrazolium salt (WST)-1 as the substrate (28). Briefly, total SOD activity of each immunoprecipitated protein sample (normalized to that of the control group) was measured based on the inhibition of the rate of WST-1 reduction. Potassium cyanide was added to the lysate during the assay to inhibit SOD1/SOD3, and 450 nm absorbance was read on a SpectraMax M5 Microplate Reader. Relative SOD2 activity (compared with that of the control group) is shown.

### Small-Intestine Epithelial-Cell Scraping and Isolation

We carefully removed 10 cm of the ileum 3 cm distal to the ligament of Treitz, placed the segment on ice, rinsed it thoroughly with normal saline, refilled it with 10 mmol/L dithiothreitol in enterocyte isolation buffer (17 mmol/L HEPES, 25 mmol/L NaHCO_3_ in PBS, pH 7.4), and tied off the segment at both ends. The segment was gently massaged to remove mucus, and after draining the luminal contents, the segment was opened longitudinally to expose the intestinal mucosa. The mucosal layer was harvested through gentle scraping, and the isolated enterocytes were used for mitochondrial function analysis (34).

### Mitochondrial Morphology

All mice (6/group) were sacrificed and small-intestine tissue was collected for morphological examination and protein extraction. Morphological changes in enterocyte mitochondria were assessed using transmission electron microscopy. Small-intestine tissues were fixed with 2.5% glutaraldehyde, stained with cacodylate-buffered osmium tetroxide, sectioned, and examined under an electron microscope (H-7500; Hitachi) (12).

### Mitochondrial-Function Evaluation

Isolated enterocytes were used for detecting mitochondrial permeability transition pore (mPTP) opening and cellular ATP levels, as described previously (9). Fluorescence intensity reflected the status of mPTP opening. ATP content was determined using an assay that measures ATP through the energy-dependent luciferase/luciferin reaction, and provides information on cell viability. The test was performed according to manufacturer instructions: 100 μL of CellTiter-Glo was added to cell suspensions (100 μL) containing 10,000 isolated cells/well of standard opaque-walled 96-well plates; after incubation at room temperature for 10 min, luminescence was recorded in a SpectraMax M5 Microplate Reader.

### Morphological Observation and Autophagy Quantification

The salient electron microscopic attribute of autophagy is the presence of phospholipid bilayers or cytoplasmic material within lysosomes; more strictly, autophagic vacuoles/autophagolysosomes are part of a morphologic spectrum that include organelles in various states of degradation within membrane-bound vacuoles or lysosomes. Thus, autophagosomes were counted under transmission electron microscopy.

The information about mouse origin and breeding, the other methods of survival time measurement, FITC-dextran assay, quantification of bacterial (CFU), diamine oxidase (DAO) determination, western blotting, deacetylase-activity determination, GSH content, GSH/GSSG ratio, and CAT activity, and apoptosis assay are presented in the supporting information.

### Statistical Analysis

Data are presented as means ± SEM of n determinations (n: number of animals studied). Statistical evaluation was performed using analysis of variance (ANOVA) followed by Bonferroni multiple-comparison test. Fisher’s exact test was used for determining significant differences in survival rate between control and drug-treated groups. *P* < 0.05 was considered significant.

## Results

### Melatonin Administration Increases Survival Time and Alleviates Intestinal Barrier Dysfunction in Septic Mice

We investigated melatonin’s protective effect on multiple organs after sepsis by administering different doses of melatonin (**SFigure 2A**). Melatonin markedly enhanced both survival time and rate in a 24-h observation window, with the effect on survival rate saturating at a dose of 30 mg/kg. Thus, 30 mg/kg melatonin was applied in subsequent analyses. Next, we examined melatonin’s effect on lung, liver, kidney, and small intestine in mice with CLP-induced sepsis by assessing five serological markers; ALT/AST/Tbil/Bun/CREA levels were increased after CLP, but were decreased by melatonin administration (**SFigures 2B–F**). Moreover, CLP-treated mice exhibited severe multiple-organ damage (lung/liver/kidney/small intestine), as revealed by increased pathological injury scores (**SFigure 2G**); notably, melatonin significantly alleviated lung and small-intestine tissue damage and slightly reduced liver and kidney injury, as indicated by decreased pathological injury scores (**SFigures 2H–J**). In the small intestine, melatonin reduced the extent of tissue structural damage and inflammatory response at 8 h post-CLP, with a reduction in the Chiu score (**SFigure 2K**).

To eliminate model bias, we also used LPS injection to mimic the sepsis model and reconfirmed melatonin’s protective effect; melatonin administration again significantly attenuated lung and small-intestine tissue damage, slightly reduced liver and kidney injury, and lowered serum inflammatory cytokines (**SFigure S3**). These data collectively indicate that, besides the lungs, the small intestine is the main organ wherein melatonin exerts its protective effect against sepsis.

**Figure 1:**
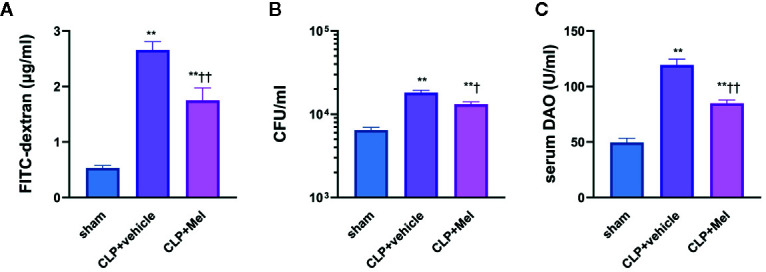
Effect of melatonin on intestinal barrier dysfunction at 8 h after CLP treatment. The mice were sacrificed through cervical dislocation for blood-sample and tissue extraction, and were prepared for the determination of intestinal barrier related indexes. **(A)** Fluorometric quantification of FITC concentration from whole-blood samples. **(B)** Quantification of colony-forming units (CFU) on blood agar plates seeded with small intestine samples. **(C)** Measurement of diamine oxidase (DAO) concentration in serum. The blood sample or small intestine sample were obtained 8 h after the CLP operation. N = 4–8. Data represent means ± SEM. ^**^ *P* < 0.01 versus sham group; ^†^ *P* < 0.05, ^††^ *P* < 0.01 versus CLP+vehicle group. CLP, cecal ligation and puncture; Mel, melatonin; FITC, fluorescein isothiocyanate; CFU, colony-forming units; DAO, diamine oxidase.

We speculate that the effect of melatonin in reducing intestinal injury is related to intestinal barrier protection and we carried out *in vivo* intestinal permeability studies, which included FITC-dextran assay and quantification of bacterial (CFU) in small intestinal tissue, and Diamine oxidase (DAO) level determination in serum. FITC concentration, CFU, and serum DAO were considerably elevated following CLP post 8h. Of note, melatonin administration significantly attenuated intestinal mucosal barrier dysfunction evidenced by reduced level of concentration, CFU, and serum DAO (**Figure 1**).

### Melatonin Attenuates Sepsis-Induced Small-Intestine Injury by Upregulating SIRT1/3 Rather Than Other SIRTs

**Figure 2:**
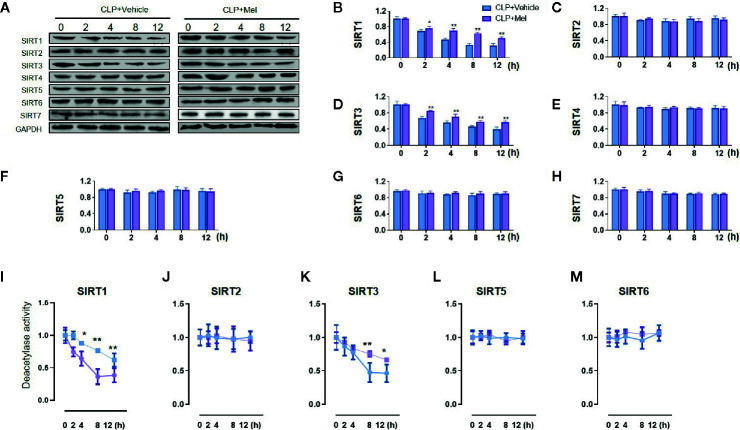
Effect of melatonin on Sirtuin protein expression and deacetylase activity in small intestine at 8 h after CLP treatment. The mice were sacrificed through cervical dislocation at 8 h after CLP treatment for tissue extraction. The samples from the intestine were collected and prepared for the determination of Sirtuin protein expression and deacetylase activity. **(A)** Representative western blots showing SIRT1–7 staining. GAPDH: internal reference. **(B–H)** Densitometric analysis of SIRT1–7. Data are presented as fold-change over CLP+vehicle group at the same time point. N = 3. Data represent means ± SEM. ^**^ *P* < 0.01 versus CLP+vehicle group. **(I–M)** Relative deacetylase activity of SIRT1/2/3/5/6. N = 3. Data represent means ± SEM. ^*^ *P* < 0.05, ^**^ *P* < 0.01 versus CLP+vehicle group at the same time point. CLP, cecal ligation and puncture; Mel, melatonin; GAPDH, glyceraldehyde-3-phosphate dehydrogenase.

We determined SIRT1–7 protein expression (**Figures 2A–H**) and the deacetylase activity (**Figures 2I–M**) at 0–24 h post-CLP. Whereas both expression and activity of SIRT1/3 were markedly decreased at corresponding time points, neither expression nor activity of SIRT2/4/6/7 was diminished. As expected, melatonin potently restored SIRT1/3 expression and particularly activity but did not affect SIRT2/4/5/6/7 expression and SIRT2/5/6 activity (**Figure 2**). Because melatonin’s effect was most notable at 8 h post-CLP, this time point was selected for further analyses.

**Figure 3:**
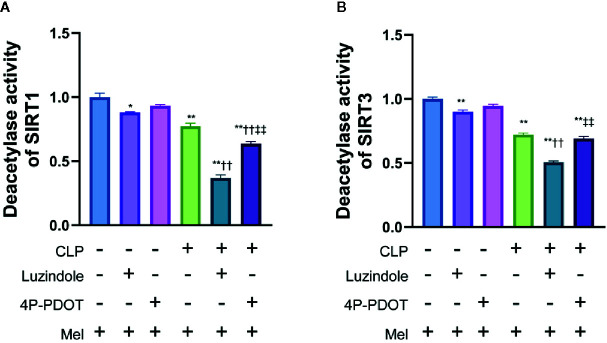
Effect of melatonin on deacetylase activity of SIRT1 and SIRT3 relying on MT1 and/or MT3 melatonin receptors. The mice were sacrificed through cervical dislocation at 8 h after CLP treatment for tissue extraction. The samples from the intestine were collected and prepared for the determination of Sirtuin protein expression and deacetylase activity. Luzindole was i.p. as non-selective MT1/MT2 receptor antagonist at a dose of 40 mg/kg dissolved in 10% DMSO 30min before CLP (diluted with edible oil based on the previous literature and our preliminary experiment), and 4P-PDOT was i.p. as selective MT2 receptor antagonist at a dose of 1mg/kg 30min before CLP. **(A)** Effect of melatonin on deacetylase activity of SIRT1. **(B)** Effect of melatonin on deacetylase activity of SIRT1. N = 4. Data represent means ± SEM. ^*^ *P* < 0.05, ^**^ *P* < 0.01 versus vehicle group; ^††^ *P* < 0.01 versus CLP+Mel group; ^‡‡^ *P* < 0.01 versus CLP+Mel+Luzindole group. CLP, cecal ligation and puncture; Mel, melatonin.

In order to clarify the relationship between the activity of melatonin and SIRT1/3, we conducted research on the commonly reported melatonin receptors MT1 and MT2 based on the literature (35). Competitive melatonin receptor antagonist luzindole (MT1/MT2-nonselective) and 4-phenyl-2-propionamidotetralin (4P-PDOT, MT2-selective) was i.p. injection at a dose of 40 mg/kg and 1.0 mg/kg dissolved in 10% DMSO (diluted with edible oil; the dose of luzindole (36) and 4P-PDOT (37) was chosen based on previous literature and our preliminary experiment. The benefit effect of melatonin on SIRT1 activation was considerably blocked by luzindole (36) and slightly blocked by 4P-PDOT in small intestine 8 h following sepsis (**Figure 3A**). In addition, the benefit effect of melatonin on SIRT3 activation was considerably blocked by MT1/MT2-nonselective antagonist luzindole but was not blocked by MT2-selective antagonist 4P-PDOT in small intestine 8 h following sepsis (**Figure 3B**).

### Melatonin Attenuates Sepsis-Induced Small-Intestine Injury Partially Through SIRT1-Activation-Mediated Inflammation Inhibition

**Figure 4:**
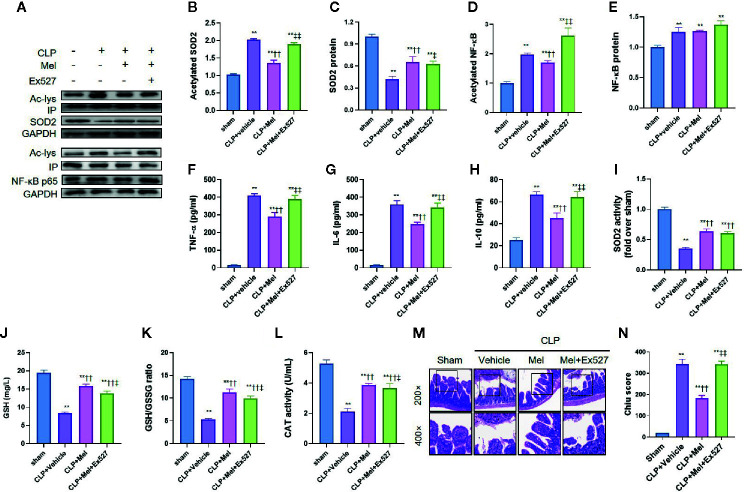
Effect of melatonin on inflammation and oxidative stress at 8 h after CLP treatment. The mice were sacrificed through cervical dislocation at 8 h after CLP treatment for blood-sample and tissue extraction and for the determination of inflammation and oxidative stress. **(A)** Representative western blots showing staining for SOD2 and NF-κB and acetylated SOD2 and NF-κB. **(B–E)** Densitometric analysis of acetylated SOD2 **(B)**, SOD2 **(C)**, acetylated NF-κB **(D)**, and NF-κB **(E)**. Data are shown as fold-change over sham group and represent means ± SEM. N = 3. **(F–H)** Measurement of serum inflammatory cytokines: F: TNF-α; G: IL-6; H: IL-10. N = 6. **(I)** SOD2 activity determination. N = 6. **(J–L)** Determination of oxidative-stress-related indexes in small-intestine homogenates: J: GSH; K: GSH/GSSG; L: CAT. N = 6. **(M, N)** Small-intestine histopathology and Chiu score. M: Representative images of small intestine after hematoxylin and eosin staining; upper and lower panels: 200× and 400× magnification images of pathological sections, respectively. N: Quantitative scores of small intestines, based on the standard Chiu score. In each tissue sample, 10 random fields were scored, and the mean value was calculated for statistical analysis. Data represent means ± SEM. ^**^ *P* < 0.01 versus sham group; ^††^ *P* < 0.01 versus CLP+vehicle group; ^‡^ *P* < 0.05, ^‡‡^ *P* < 0.01 versus CLP+Mel group. CLP, cecal ligation and puncture; Mel, melatonin; IP, immunoprecipitation; IL, interleukin; GSH, reduced glutathione; GSSG, oxidized glutathione; CAT, catalase; GAPDH, glyceraldehyde-3-phosphate dehydrogenase.

To examine melatonin’s effect on SIRT1 and the downstream signaling, we used the SIRT1-specific inhibitor Ex527. Sepsis induction reduced NF-κB and SOD2 protein expression but increased their acetylation (**Figures 4A–E**), and this was accompanied by an increase in proinflammatory cytokines (TNF-α/IL-6/IL-10; **Figures 4F–H**) and oxidative stress (indicated by reduced SOD2 activity, GSH content, GSH/GSSG ratio, and CAT activity; **Figures 4I–L**). As expected, melatonin reduced NF-κB/SOD2 acetylation, increased SOD2 activity, and lowered TNF-α/IL-6/IL-6 and oxidative-stress levels. Moreover, melatonin substantially ameliorated small-intestine injury, as indicated by reduced histopathological staining and Chiu score (**Figures 4M, N**). Intriguingly, melatonin’s inhibition of inflammation, but not oxidative stress, was potently blocked by Ex527, as revealed by the re-elevation of proinflammatory cytokines and intestinal-injury score; notably, Ex527 did not block melatonin’s effect on SOD2 acetylation/activity and other oxidative-stress indexes (**Figure 3**).

### Melatonin’s Beneficial Effect on Sepsis-Induced Small-Intestine Injury Also Depends on SIRT3 Activation

**Figure 5:**
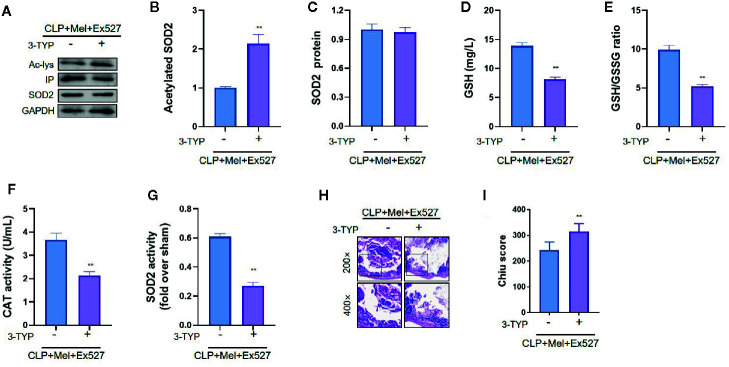
Effect of SIRT3 inhibition on SOD2 protein expression, SOD2 activity, oxidative-stress indexes, and histopathology of small intestine after CLP treatment. The mice were sacrificed through cervical dislocation at 8 h after CLP treatment for blood-sample and tissue extraction. The samples from the intestine were collected and prepared. **(A)** Representative western blots showing staining for SOD2 and acetylated SOD2. **(B, C)** Densitometric analysis of acetylated-SOD2 levels and SOD2 protein expression. N = 3. **(D)** SOD2 activity determination. N = 6. **(E–G)** Determination of oxidative-stress-related indexes in small-intestine homogenates: E: GSH; F: GSH/GSSG; G: CAT. N = 6. **(H, I)** Small-intestine histopathology and Chiu score. H: Representative images of small intestines after hematoxylin and eosin staining; upper and lower panels: 200× and 400× magnification images of pathological sections, respectively. I: Quantitative score of small intestines, based on the standard Chiu score. In each tissue sample, 10 random fields were scored, and the mean value was calculated for statistical analysis. Data are presented as fold-change over CLP+Mel+Ex527 group and represent means ± SEM. ^**^ *P* < 0.01. CLP, cecal ligation and puncture; Mel, melatonin; IP, immunoprecipitation; Ac, acetylated; 3-TYP, 3-(1H-1,2,3-triazol-4-yl) pyridine; GSH, reduced glutathione; GSSG, oxidized glutathione; CAT, catalase; GAPDH, glyceraldehyde-3-phosphate dehydrogenase.

Because oxidative stress attenuation by melatonin did not rely on SIRT1 activation, we tested whether melatonin’s antioxidative effect depends on SIRT3: addition of the SIRT3-selective chemical inhibitor 3-TYP considerably increased oxidative-stress indexes—enhanced SOD2 acetylation, reduced SOD2 activity, and decreased GSH content, GSH/GSSG ratio, and CAT level (**Figures 5A, B, D–G**)—and aggravated small-intestine injury (increased pathology score; **Figures 5H, I**), but did not lower SOD2 protein content (**Figure 5C**).

**Figure 6:**
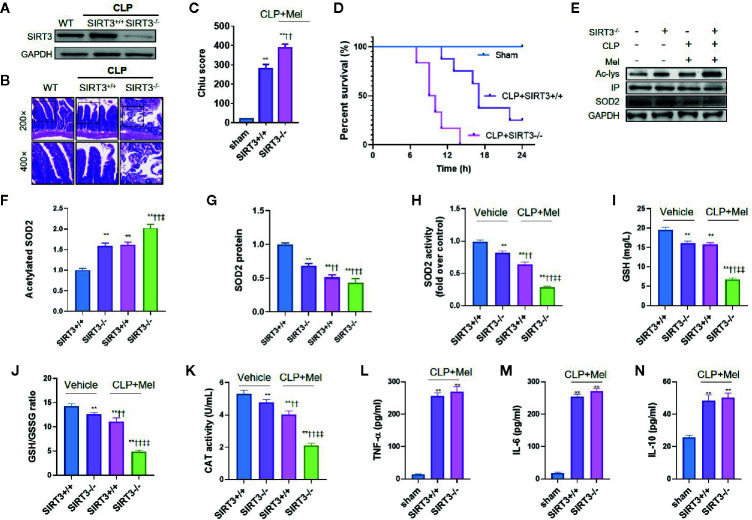
SIRT3 conditional knockout blocks melatonin effect on oxidative-stress inhibition but not inflammation inhibition after CLP treatment. The mice were sacrificed through cervical dislocation at 8 h after CLP treatment for tissue extraction. The samples from the intestine were collected and prepared. **(A)** Confirmation through western blotting of intestinal SIRT3 conditional knockout. Representative bands are shown. N = 3. **(B, C)** Effect of SIRT3 conditional knockout (SIRT3^-/-^) on small-intestine pathological damage and Chiu score. Representative images of small intestines after hematoxylin and eosin staining are shown; upper and lower panels: 200× and 400× magnification images of pathological sections, respectively. In each tissue sample, 10 random fields were scored, and the mean value was calculated for statistical analysis. ^**^ *P* < 0.01 versus sham group; ^††^ *P* < 0.01 versus SIRT3^+/+^+vehicle group. **(D)** Effect of SIRT3 conditional knockout on survival time of septic mice. N = 8. **(E)** Representative western blots showing staining for SOD2 and acetylated SOD2 after CLP treatment. **(F, G)** Densitometric analysis of acetylated SOD2 **(F)** and SOD2 protein **(G)**. N = 3. ^**^ *P* < 0.01 versus SIRT3^+/+^+vehicle group. ^††^ *P* < 0.01 versus SIRT3^-/-^+vehicle roup ^‡‡^ *P* < 0.05, ^‡‡^ *P* < 0.01 versus SIRT3^+/+^+vehicle+Mel group. **(H)** Effect of SIRT3 conditional knockout on SOD2 activity. N = 6. **(I–K)** Effect of SIRT3 conditional knockout on oxidative-stress-related indexes: I: GSH content; J: GSH/GSSG ratio; K: CAT activity. N = 6. ^**^ *P* < 0.01 versus SIRT3^+/+^+vehicle group; ^††^ *P* < 0.01 versus SIRT3^-/-^+vehicle group. ^‡‡^ *P* < 0.01 versus SIRT3^+/+^+vehicle+Mel group. **(L–N)** Effect of SIRT3 conditional knockout on inflammatory cytokines: L: TNF-α; M: IL-6; N: IL-10. N = 6. ^**^ *P* < 0.01 versus sham group; ^††^ *P* < 0.01 versus SIRT3^+/+^+CLP+Mel group. Data represent means ± SEM. CLP, cecal ligation and puncture; Mel, melatonin; IP, immunoprecipitation; Ac, acetylated; IL, interleukin; GSH, reduced glutathione; GSSG, oxidized glutathione; CAT, catalase; GAPDH, glyceraldehyde-3-phosphate dehydrogenase.

We confirmed the SIRT3 effect using SIRT3 conditional-knockout mice (sirt3^flox+/+Cre+/-^; IEC-targeted) (**Figure 6A**). SIRT3-knockout mice showed increased susceptibility to sepsis, as indicated by elevated small-intestine pathology score (**Figures 6B, C**) and shortened survival time (**Figure 6D**) relative to sirt3^flox+/+Cre-/-^ mice. Consistent with the SIRT3-inhibitor results, melatonin more weakly affected oxidative stress in the small intestine of SIRT3-knockout septic mice than in sirt3^flox+/+Cre-/-^ septic mice. Specifically, SIRT3 knockout caused increased SOD2 acetylation (**Figures 6E–G**), reduced SOD2 activity (**Figure 6H**), and lowered GSH content, GSH/GSSG ratio, and CAT level (**Figures 6I–K**). Interestingly, SIRT3 knockout also reduced SOD2 protein expression (**Figures 5E, G**), but did not exacerbate inflammatory response as expected (unchanged TNF-α/IL-6/IL-10 levels; **Figures 6L–N**).

### Melatonin-Mediated SIRT3 Activation Ameliorates Mitochondrial Dysfunction and Promotes Autophagy

**Figure 7:**
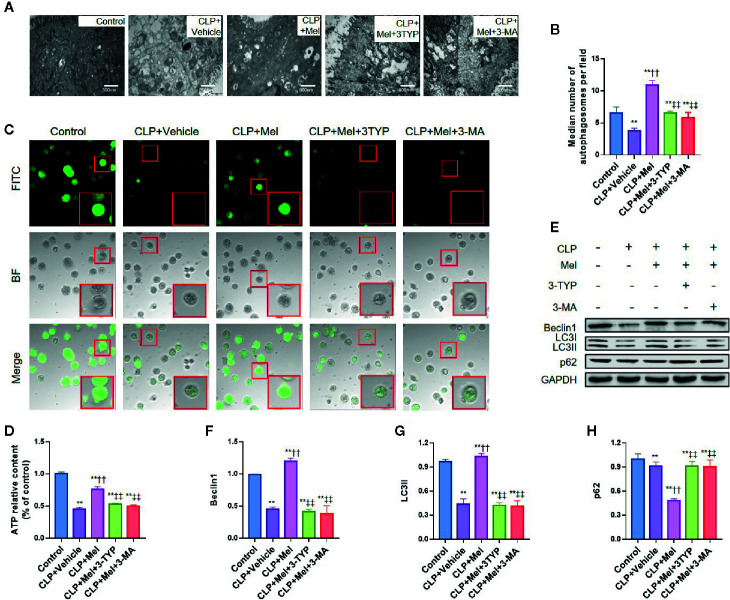
Effect of melatonin on morphology and function of mitochondria and autophagy of small-intestine epithelial cells after CLP treatment. The mice were sacrificed through cervical dislocation at 8 h after CLP treatment for tissue extraction. The samples from the intestine were collected and prepared for morphological examination and protein extraction. Isolated enterocytes were used for detecting mitochondrial permeability transition pore (mPTP) opening and cellular ATP levels. **(A)** Representative transmission electron microscopy images of mitochondria of epithelial cells in the small intestine after induction of sepsis. Healthy mitochondria (control group) present intact mitochondrial membranes and cristae. Mitochondria are swollen and harbor poorly defined cristae and show increased vacuolization after sepsis challenge in the CLP+vehicle group, and CLP+Mel+3-TYP group, and CLP+Mel+3-MA group, whereas these changes are partially prevented in the CLP+Mel group (magnification: 125,000×; inset: 500,000× magnification). **(B)** Autophagosome count, based on transmission electron microscopy. Scale bar: 500 nm. **(C)** Intensity of calcein-AM fluorescence, reflecting the extent of opening of the mitochondrial permeability transition pore (mPTP). Red boxes: typical cellular immunofluorescence. **(D)** ATP measurement in small-intestine epithelial cells. N = 6. **(E)** Representative western blots showing staining for autophagy-related proteins. **(F–H)** Densitometric analysis of autophagy-related proteins: F: Beclin1; G: LC3II; H: p62. N = 3. Data represent means ± SEM. ^**^ *P* < 0.01 versus sham group; ^††^ *P* < 0.01 versus CLP+vehicle group; ^‡‡^ *P* < 0.01 versus CLP+Mel group. CLP, cecal ligation and puncture; Mel, melatonin; BF, bright-field; FITC, fluorescein isothiocyanate; 3-TYP, 3-(1H-1,2,3-triazol-4-yl) pyridine; 3-MA, 3-methyladenine; GAPDH, glyceraldehyde-3-phosphate dehydrogenase.

SIRT3 primarily localizes in mitochondria, protects mitochondrial function, and upregulates autophagy; therefore, we suspected that melatonin-mediated SIRT3 activation in response to sepsis might involve mitochondrial protection and autophagy promotion. We examined mitochondrial morphology using electron microscopy, assessed mitochondrial function based on mPTP opening and ATP content, and evaluated autophagy upregulation based on an increase in autophagosomes and/or autophagy-related proteins Beclin1 and LC3II and decrease in p62 levels. CLP treatment caused mitochondrial dysfunction, as shown by mitochondrial swelling with a disappearance of mitochondrial cristae (**Figure 7A**), increased mPTP opening (**Figure 7B**), and decreased ATP content (**Figure 7C**), whereas melatonin alleviated mitochondrial dysfunction: mitochondrial cristae were retained, mPTP opening was diminished, and ATP content was preserved. Notably, melatonin’s protective effect was potently counteracted by SIRT3 inhibition with 3-TYP or by autophagy inhibition with 3-methyladenine (3-MA) (**Figures 7A–C**). Furthermore, CLP inhibited autophagy induction, as shown by decreased autophagosomes (**Figure 7D**), reduced Beclin1 and LC3II expression, and increased p62 content (**Figures 7E–H**), but melatonin promoted autophagy (increased autophagolysosomes, elevated Beclin1/LC3II, decreased p62) and this was strongly counteracted by 3-TYP or 3-MA (**Figure 7**).

## Discussion

We confirmed that melatonin treatment alleviates small-intestine injury *via* protection of intestinal barrier in mouse sepsis models. Mechanically, melatonin attenuated sepsis-induced small-intestine injury through SIRT1/3 activation. Melatonin upregulated SIRT1-induced NF-κB deacetylation and subsequently reduced inflammatory cytokines, as well as upregulated SIRT3-induced SOD2 deacetylation, reduced oxidative stress, elevated autophagy, and preserved mitochondrial function. To the best of our knowledge, this is the first elucidation of melatonin-mediated SIRT3 activation in protection against sepsis-induced small-intestine injury.

Only a few studies have reported melatonin’s effect on sepsis-induced small-intestine injury. Melatonin reversed CLP-induced ileal dysfunction in rats, and its protection of small-intestine tissue against sepsis relied on oxidative-damage inhibition (38, 39). Moreover, melatonin’s beneficial effect against endotoxemia-induced intestinal injury in newborn rats was depended on reduced apoptosis (29). Here, we monitored plasma melatonin levels under increasing melatonin dosage and investigated how melatonin affects multiple organs (lung/liver/kidney/small intestine) and systemic inflammatory response. Exogenous melatonin (applied i.p.) increased plasma melatonin levels at 8 h post-sepsis induction (**SFigure 4**). Because endogenous melatonin secretion is generally accepted to be affected by circadian rhythm (40), we constructed a mouse sepsis model featuring almost the same timepoints to avoid potential interference; consistent with a previous work, melatonin relieved small-intestine injury (jejunum/ileum), and notably preserved mitochondrial function and elevated autophagy in small-intestine epithelial cells in response to sepsis, suggesting a previously unknown mechanism underlying melatonin’s protection against sepsis-induced small-intestine injury. In this study, melatonin reduced the level of FITC-dextran in serum, CFU in small intestine tissue and DAO concentration in serum, suggesting that the protective effect of melatonin against intestinal injury lies in maintaining the intestinal barrier function, reducing intestinal exudation and possible translocation of intestinal bacteria.

Melatonin’s effect on SIRT-family members has been extensively investigated recently (11). SIRTs function mainly in epigenetic modification (deacetylation) and posttranscriptional modification, which might represent the key mechanism underlying melatonin’s protection against sepsis (11). Melatonin reportedly alleviates sepsis-induced multiple-organ injury, with the underlying mechanism mainly relying on SIRT1 activation. In a sepsis-induced brain-injury model, melatonin-mediated SIRT1 upregulation caused FoxO1 (forkhead box O1), p53, and NF-κB deacetylation and correspondingly inhibited oxidative stress, apoptosis, and inflammatory response (13). Moreover, melatonin reduced liver injury (16) and cardiac dysfunction (10) after sepsis through SIRT1 upregulation. Our study confirmed melatonin’s role in SIRT1 protein/activity upregulation in sepsis-challenged small-intestine tissue. We propose that melatonin mainly affects SIRT1 deacetylase activity, because melatonin’s anti-inflammatory effect was potently blocked by SIRT1-selective inhibitor Ex527. Moreover, SIRT1 activity was elevated to a greater extent than SIRT1 expression after melatonin treatment. In contrast to a previous study (13), we did not detect further oxidative stress elevation after SIRT1 inhibition, indicating that melatonin’s role in oxidative-stress alleviation does not depend completely on SIRT1 upregulation. Moreover, we collected small-intestine samples as early as 8 h post-CLP, which might not represent an optimal time of SIRT1-mediated oxidative-stress suppression.

Besides SIRT1, SIRT3 is a key melatonin target verified in various animal models: SIRT3 localizes primarily to mitochondria and protects against oxidative-stress-related diseases (41), and melatonin can upregulate SIRT3 to attenuate myocardial ischemia–reperfusion injury (17, 42) through oxidative-stress inhibition. Moreover, melatonin notably mitigated adverse left ventricle remodeling and alleviated cardiac dysfunction in diabetic cardiomyopathy through SIRT3 signaling (43). SIRT3 activation causes deacetylation of specific targets, such as the mitochondrial antioxidative enzyme SOD2 (41), and signaling downstream from SOD2 deacetylated by melatonin-upregulated SIRT3 alleviates NaF-induced hepatotoxicity (19) and Cd-induced hepatotoxicity (44). We found that oxidative-stress alleviation by melatonin is closely related to SIRT3 activation in sepsis-induced small-intestine injury. To elucidate how melatonin-regulated SIRT3 affects oxidative stress, we applied a SIRT3-specific inhibitor and found that melatonin’s anti-oxidative-stress effect depends on SIRT3 activation. We also confirmed this mechanism in a sepsis model developed using SIRT3 conditional-knockout mice. We further found that melatonin slightly increased SOD2 protein expression, which might be due to SIRT3-regulated DNA-binding activity of FoxO3a (19). Our study has highlighted—for the first time—a role of melatonin in SIRT3-SOD2 activation in response to sepsis-induced small-intestine injury.

Intriguingly, melatonin-mediated SIRT3 upregulation not only suppressed oxidative stress, but also preserved mitochondrial function and upregulated autophagy. Melatonin targets mitochondria and functions as an apex antioxidant (45), and besides being taken up by cells from the circulation, melatonin might be produced in mitochondria (45). Melatonin can attenuate TNF-α-mediated hepatocyte damage through SIRT3-mediated mitochondrial-stress inhibition (18), and counteracts mitochondrial dysfunction in the skeletal muscle of septic mice (46). Furthermore, SIRT3-mediated mitophagy protects tumor cells against apoptosis under hypoxia (46). Melatonin increases electron transport chain activity and thus enhances mitochondrial respiration and ATP synthesis under normal and stressful conditions; accordingly, melatonin reduces the harmful reduction in mitochondrial membrane potential that could trigger mitochondrial transition-pore opening and the apoptosis cascade (47). Consistent with previous studies, we confirmed that melatonin restored mitochondrial morphology and function in sepsis-challenged small-intestine epithelial cells. Notably, melatonin’s protective effect on mitochondria was reversed by SIRT3 inhibition, indicating a critical role of SIRT3. However, in pathological examination of small-intestine tissue, we did not detect IEC apoptosis, which might be due to the sepsis stimulation being at an early stage (**SFigure 5**). However, we believe that the observed mitochondrial damage is, as generally accepted, an indicator of subsequent apoptosis.

Melatonin was first reported to suppress Cd-induced autophagic cell death by enhancing SIRT3 activity *in vivo* (44). However, melatonin administration was also found to promote autophagic flux, indicated by elevated LC3II and lowered p62 expression, in a mouse model of diabetic cardiomyopathy, and this was associated with SIRT3 signaling (43). We confirmed here that melatonin elevated autophagy by examining cellular morphology and autophagy-related proteins. Unexpectedly, melatonin’s effect on autophagy was found to depend, at least partially, on SIRT3 activation. Many literatures have reported the role of SIRT3 on autophagy activation in non-sepsis models (48–52). Enhanced SIRT3 activity could decrease SOD2 acetylation, inhibit the production of mitochondrial reactive oxygen species, and suppress iron loading-induced autophagy (48). Knockdown of SIRT3 resulted in decreased autophagy in peritoneal macrophages and RAW 264.7 cells from stress-induced mice (51). Mechanically, SIRT3 upregulation protects neurons against cerebral ischemia *via* AMPK-mTOR pathway (50). In addition, SIRT3 coordinates mitochondrial function and autophagy activation to promote anti-mycobacterial responses through peroxisome proliferator activated receptor alpha (PPARA) (49). To date, the role of SIRT3 activation on autophagy in sepsis is rarely reported. A study suggests that SIRT3 protects against CLP-induced AKI by inducing autophagy through regulation of the AMPK/mTOR pathway (53). In this study, we confirmed that melatonin upregulates autophagy by activating SIRT3. On the one hand, we speculate that the mechanism is related to the indirect activation of autophagy by oxidative stress inhibition and protection of mitochondria. On the other hand, we speculate that SIRT3 may directly activate autophagy through deacetylation modification of autophagy-related proteins such as ATG5, which have been confirmed in peripheral blood monocytes (52). Although we did not further investigate the mechanism by which melatonin enhances autophagy, our results emphasize the role of SIRT3 activation in melatonin-dependent protection against sepsis-induced small-intestine injury.

Our study’s limitations are as follows. First, we investigated melatonin’s effect only on the early stage of sepsis-induced multiple-organ injury, when inflammatory cytokines are sharply increased; inflammation level and immune status change dynamically after sepsis and must be monitored to test how melatonin affects different stages of sepsis. Second, given the short observation time and our aim here to magnify melatonin’s therapeutic effect, we administered only two doses of melatonin, 10 min before CLP surgery or LPS injection and 30 min after sepsis induction; this is inconsistent with the biological rhythm and timing of sepsis treatment. We believe that repeated melatonin administration (4–6 h dosing intervals) might represent a potential future adjuvant treatment for sepsis. Third, the SIRT3 conditional knockout was induced in the intestine, which includes the small and large intestines; thus, we cannot eliminate the possibility that melatonin-rich colon (54) also affects sepsis-induced bowel injury prognosis. Fourth, we believe the mechanism of melatonin-mediated SIRT3 activation in ameliorating sepsis is not limited to SOD2 deacetylation and warrants comprehensive investigation in the future. Nevertheless, our study has emphasized the key role of SIRT3 in melatonin-mediated protection against sepsis-induced small-intestine injury.

Our study reveals that melatonin alleviates sepsis-induced small-intestine injury by upregulating SIRT1 and SIRT3. Melatonin enhances the activity and expression of SIRT1 and subsequent NF-κB deacetylation, thus inhibiting inflammation. Melatonin also independently upregulates the activity and expression of SIRT3 in the mitochondria and hence triggers SOD2 deacetylation, thus inhibiting oxidative stress, protecting mitochondrial function, inducting autophagy. Our research provides further mechanistic support for melatonin for the treatment of clinical sepsis. Further studies are warranted to confirm the therapeutic effects and benefits of melatonin in patients with sepsis-induced small intestine injury.

## Data Availability Statement 

The original contributions presented in the study are included in the article/**Supplementary Material**. Further inquiries can be directed to the corresponding author.

## Ethics Statement

The animal study was reviewed and approved by Committee on Ethics in Animal Experiments of Southern Medical University.

## Author Contributions

SX, LL, JW, SA, HF, and FW performed the research. ZZ and ZC designed the research study. YH and SX contributed essential reagents or tools. ZZ, ZC, and YH analyzed the data. ZZ and QH wrote the paper. All authors contributed to the article and approved the submitted version.

## Funding

This work was supported by the Natural Science Foundation of China Grant 81871604; the Natural Science Foundation of Guangdong Province, China, Grants 2020A151501361 and 2017A030313590; the Outstanding Youths Development Scheme of Nanfang Hospital, Southern Medical University, Guangzhou, China, Grant 2016J011; and the President Foundation of Nanfang Hospital, Southern Medical University, Guangzhou, China, Grants 2016C026 and 2019C029.

## Conflict of Interest

The authors declare that the research was conducted in the absence of any commercial or financial relationships that could be construed as a potential conflict of interest.

## Abbreviations

ANOVA, Analysis of variance; BUN, Blood urea nitrogen; CC, Chiao CW; CLP, Cecal ligation and puncture.
