# Supplementary material for: Melatonin Attenuates Sepsis-Induced Small-Intestine Injury by Upregulating SIRT3-Mediated Oxidative-Stress Inhibition, Mitochondrial Protection, and Autophagy Induction
Source: Front Immunol. 2021 Mar 12;12:625627. doi: 10.3389/fimmu.2021.625627 (PMC8006917; doi:10.3389/fimmu.2021.625627)
Supplement: Supplementary file 1 [file DataSheet_1.pdf]

Reagents

These reagents were purchased: antibodies against SIRT1/2/3/4/5/6/7, superoxide dismutase (SOD) 2, NF-κB, Abcam; antibodies against Beclin1 and p62, ABClonal; antibodies against acetylated-lysine, glyceraldehyde-3-phosphate dehydrogenase (GAPDH), and LC3II, Cell Signaling Technology; deacetylase-activity kit for SIRT1/2/3/5/6, Cyclex; SIRT5 deacetylase-activity kit, Abcam; SOD2 activity-assay kit, Dojindo Molecular Technology Inc.; reduced-glutathione/oxidized-glutathione (GSH/GSSG) and catalase (CAT) assay kits and goat anti-rabbit secondary antibodies, Beyotime Biotech; immunoprecipitation kits, Proteintech; and membrane-permeant JC-1 dye and calcein-AM, Molecular Probes; 4-phenyl-2-propionamidotetralin (4-P-PDOT); Tocris Bioscience. All other chemicals were from Sigma.

Mouse origin and breeding

All mice were housed in specific-pathogen-free conditions under a 10/14-h light/dark cycle and provided free access to food and water. For cecal ligation and puncture (CLP) or endotoxemia experiments, 8–12-week old mice were used. C57BL/6J-background wild-type mice were originally purchased at Southern Medical University, Guangzhou, China. SIRT3$^{-/-}$ conditional-knockout mice were purchased from Shanghai Model Organisms (Pudong New Area, Shanghai, China; EMMA:06872, C57BL/6N-A^{tm1Brd}Sirt3$^{tm1a(EUCOMM)Wtsi}$/WtsiH). SIRT3$^{flox/flox}$ mice were generally crossed with Villin-cre mice to generate intestinal-epithelial-cell (IEC) SIRT3 conditional-knockout mice. All mice were further bred in our laboratory.

CLP-/endotoxemia-induced sepsis models

The CLP-induced sepsis model was developed according to general guidelines. Briefly, after mice were anesthetized through isoflurane inhalation (RWD Lifescience), a 1-cm incision was made in the abdomen and the cecum was exposed and ligated; subsequently, the cecum was punctured with a 21-gauge needle, and then the abdominal musculature and skin were closed using simple running sutures and metallic clips, respectively. Lethality was monitored for 24 h and the data were pooled.[1] At 2 h post-CLP or sham treatment, 100 μL of compound sodium chloride was gavaged.[2] The endotoxin-induced sepsis model was prepared by injecting i.p. a lethal dose of LPS (LD100 6.25 mg/kg, *Salmonella enterica*) dissolved in 100 μL of sterile phosphate-buffered saline (PBS).[2]

Melatonin was dissolved in 5% ethanol, and at 10 min before and 30 min after sepsis induction, the melatonin solution was administered at different doses (7.5/15/30/60 mg/kg bw, 50 μL/10 g, i.p.). The control (sham) groups received vehicle only at the same administration time and volume (5% ethanol, 50 μL/10 g, i.p.). SIRT1-inhibitor Ex527 and SIRT3-inhibitor 3-TYP were administered (10 and 5 mg/kg, respectively, in 100 μL of 5% ethanol, i.p.) at 30 min after sepsis induction as appropriate.[1] Animals were sacrificed through cervical dislocation at different timepoints as necessary for blood-sample and tissue extraction. The small intestine was collected and prepared for determination of quantification of bacterial, SIRT3, SOD2 and oxidative stress determination.

Survival-time measurement

Some of the animals in each group (8/group) were assigned to a subgroup for survival analyses. Survival time and prevalence at 24 h were recorded. Mice that survived for >1 d were sacrificed through cervical dislocation. Apnea for >1 min was considered to indicate death.

FITC-dextran assay

FITC-conjugated dextran (Sigma-Aldrich, St. Louis, MO) or saline was administered by oral gavage (44 mg/100 g body weight of FITC-labeled dextran) 30min prior to CLP, and the whole blood was collected by cardiac puncture. Plasma was isolated from blood samples by centrifugation for 15 min at 3000 rpm and 4°C. FITC concentration was fluorometrically quantified by emission spectrometry (Promega, Madison, WI) at 528 nm, using an excitation wavelength of 485 nm. All concentrations were measured against a standard curve of serially diluted FITC-dextran[3].

Quantification of Bacterial (CFU)

Small intestine samples were collected from mice 8 h after CLP. Tissues were homogenized 1:10 in phosphate-buffered saline (PBS) containing 0.1% Tween 20 per gram of tissue, then diluted again 1:10 in PBS. Diluted homogenates were plated on blood agar plates and incubated at 37 °C for 1–2 days. Colony forming units (CFU) were counted, and densities calculated as follows: CFU/mL= (# of colonies×sample dilution factor×serial dilution factor)/volume of culture plate (mL)[3].

Diamine oxidase (DAO) determination

The serum samples were thawed at 37 °C for 1 h, and DAO was detected with an Enzyme-linked immunosorbent assay (ELISA) kit (Mlbio, Shanghai, China), according to the manufacturer's instructions. The experiment was repeated four times[4].

Plasma melatonin determination and Serum inflammatory-cytokine measurement

Plasma melatonin levels were assessed using a melatonin ELISA kit (Wuhan Fine Biotech Co., Ltd.). Data were collected and analyzed using the manufacturer's instructions and standards. Briefly, 50-μL samples were used and then the 450-nm absorbance was measured in a microplate reader. Six samples per group were examined for statistical analysis. Frozen serum was thawed on ice and TNF-α, IL-6, and IL-10 levels were measured using ELISA kits, as per manufacturer instructions (Proteintech Group, Inc.).

Western blotting

From small-intestine samples, proteins were extracted at 8 h post-operation, and after concentration measurement (BCA Protein Assay kit; Beyotime), proteins were separated using SDS-PAGE and transferred to PVDF membranes (Millipore), which were blocked with 5% nonfat milk in 0.05% Tween 20 (TBST) (room temperature) and then incubated (overnight, 4°C) with primary antibodies (1:1000) against SIRT1/2/3/4/5/6/7, SOD2, NF-κB, p62, LC3II, Beclin1, and GAPDH. Subsequently, the membranes were washed thrice with TBST (10 min each), incubated with appropriate horseradish peroxidase-conjugated secondary antibodies (1:5000, room temperature, 1.5 h), and washed thrice with TBST. Protein bands were detected using a Bio-Rad imaging system and quantified using Quantity One software package.

Deacetylase-activity determination

SIRT1/2/3/5/6 deacetylase activity was detected using commercial deacetylase fluorometric assay kits, based on referring to manufacturer instructions and our previous methods.[5,6] Briefly, small-intestine tissue samples (25 mg) were homogenized in 500 μL of immunoprecipitation buffer, and after SIRT1/2/3/5/6 immunoprecipitation, the 50-μL final reaction mixtures contained 50 mmol/L Tris-HCl (pH 8.8), 4 mmol/L $MgCl_2$, 0.5 mmol/L dithiothreitol, 0.25 mA/mL lysyl endopeptidase, 1 μmol/L trichostatin A, 200 μmol/L NAD^+, and 5 μL of extraction buffer. Fluorescence intensity

was measured at 340/360 and 480/500 nm for SIRT1/3 and SIRT2/5/6, respectively, on a SpectraMax M5 Microplate Reader (Molecular Devices). Activity is presented as a relative value compared with that of the control group.

Competitive melatonin receptor antagonist luzindole (MT1/MT2-nonselective) and 4-phenyl-2-propionamidotetralin (4P-PDOT, MT2-selective) was i.p. injection at a dose of 40 mg/kg and 1.0mg/kg dissolved in 10% DMSO (diluted with edible oil; the dose of luzindole[7] and 4P-PDOT[8] was chosen based on the previous literature and our preliminary experiment.

GSH content, GSH/GSSG ratio, and CAT activity

Small-intestine tissue samples were minced, homogenized, and centrifuged (12,000 × *g*, 20 min), and GSH content, GSSG/GSH ratio, and CAT activity in isolated small intestinal epithelial cells were evaluated using kits, according manufacturer instructions and standard methods. Briefly, 200 μL of homogenized tissue samples were mixed with the kit reagent and processed in boiled water and then ice, as per the instructions. Subsequently, 520-nm absorbance was measured for GSH and GSSG on a microplate reader, and the enzyme concentrations were calculated. For CAT, 520-nm absorbance was obtained on a UV spectrophotometer, and CAT values relative to the control group were calculated.

Apoptosis assay

TUNEL staining was performed using a Promega apoptosis-detection kit to identify apoptotic cells in small-intestine tissue, as per manufacturer instructions. For TUNEL immunofluorescence analysis, fluorescein isothiocyanate (FITC; green) was used. Coverslips were mounted on glass slides with 4′,6-diamidino-2-phenylindole (DAPI; blue) and imaged under a confocal microscope (LSM 780; Carl Zeiss). TUNEL-positive apoptotic cells were detected as cells that fluoresced green and blue. Both TUNEL-positive and DAPI-positive cells were counted in 10 random high-power fields (HPFs; 300 cells each). Data are expressed as number of apoptotic cells/HPF (400× magnification).

Reference

1. Wei S, Gao Y, Dai X, et al. SIRT1-mediated HMGB1 deacetylation suppresses sepsis-associated acute kidney injury. *American Journal of Physiology-Renal Physiology.* 2019-01-01 2019;316:F20-F31.
2. Ozdemir D, Uysal N, Tugyan K, et al. The effect of melatonin on endotoxemia-induced intestinal apoptosis and oxidative stress in infant rats. *Intensive Care Medicine.* 2007-02-26 2007;33:511-516.
3. Kang M, Mischel RA, Bhave S, et al. The effect of gut microbiome on tolerance to morphine mediated antinociception in mice. *Sci Rep.* Feb 17 2017;7:42658.
4. Cao Y, Chen Q, Wang Z, et al. PLK1 protects against sepsis-induced intestinal barrier dysfunction. *Sci Rep.* Jan 18 2018;8:1055.
5. Zeng Z, Chen Z, Xu S, et al. Polydatin Protecting Kidneys against Hemorrhagic Shock-Induced Mitochondrial Dysfunction via SIRT1 Activation and p53 Deacetylation. *Oxid Med Cell Longev.* 2016-01-20 2016;2016:1737185.
6. Zeng Z, Yang Y, Dai X, et al. Polydatin ameliorates injury to the small intestine induced by hemorrhagic shock via SIRT3 activation-mediated mitochondrial protection. *Expert Opin Ther Targets.* 2016-06-01 2016;20:645-652.
7. Luo Y, Yang Y, Shen Y, et al. Luzindole attenuates LPS/d-galactosamine-induced acute hepatitis in mice. *Innate Immun.* May 2020;26:319-327.
8. Shin EJ, Chung YH, Le HL, et al. Melatonin attenuates memory impairment induced by Klotho gene deficiency via interactive signaling between MT2 receptor, ERK, and Nrf2-related antioxidant potential. *Int J Neuropsychopharmacol.* Dec 30 2014;18.
