# Supplementary figures and images for: Melatonin Attenuates Sepsis-Induced Small-Intestine Injury by Upregulating SIRT3-Mediated Oxidative-Stress Inhibition, Mitochondrial Protection, and Autophagy Induction
Source: Front Immunol. 2021 Mar 12;12:625627. doi: 10.3389/fimmu.2021.625627 (PMC8006917; doi:10.3389/fimmu.2021.625627)

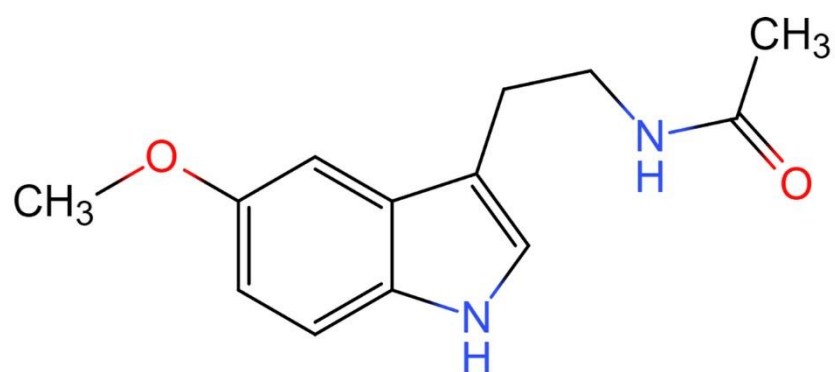

**Figure 1.** Molecular structure of melatonin.

Supplement: Supplementary file 2 [file DataSheet_2.pdf]
