# Supplementary material for: Melatonin Attenuates Sepsis-Induced Small-Intestine Injury by Upregulating SIRT3-Mediated Oxidative-Stress Inhibition, Mitochondrial Protection, and Autophagy Induction
Source: Front Immunol. 2021 Mar 12;12:625627. doi: 10.3389/fimmu.2021.625627 (PMC8006917; doi:10.3389/fimmu.2021.625627)
Supplement: Supplementary file 3 [file DataSheet_3.pdf]

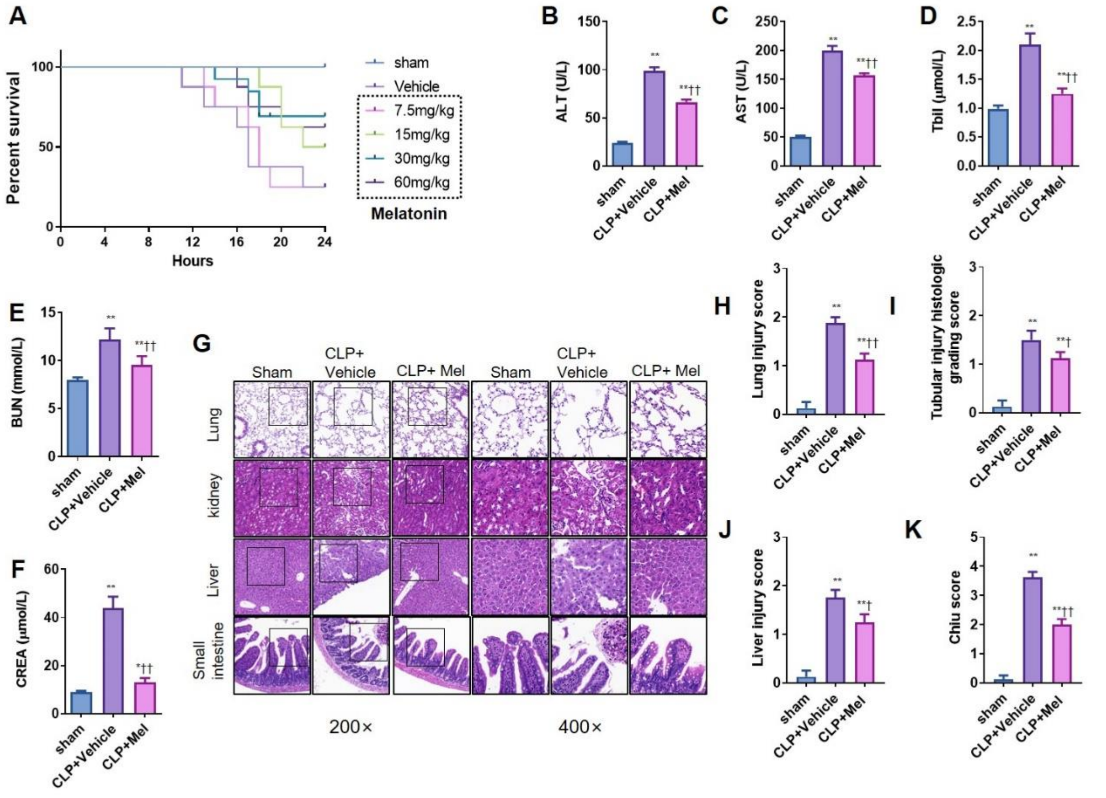

SFigure 2. Effect of melatonin on survival time, multiple-organ function, and histopathological-injury score. (A) Effect of different doses of melatonin on survival time of CLP mice. N = 6. (B-F) Serum indexes of liver and kidney function (assessed at 8 h after CLP surgery): B: alanine aminotransferase (ALT); C: aspartate aminotransferase (AST); D: total bilirubin (Tbil); E: blood urea nitrogen (BUN); F: creatinine (CREA). N = 6. (G) Representative images of lung, kidney, liver, and small-intestine tissues after hematoxylin and eosin staining; left and right panels: 200× and 400× magnification images of pathological sections, respectively. (H-K) Quantitative scoring standards of multiple organs: H: lung; I: kidney; J: liver; K: small intestine. The standards for pathohistological scores and the degree of multiple-organ injury were evaluated under a microscope by two senior technicians blinded to the treatment protocol. In each tissue sample, 10 random fields were scored, and the mean value was calculated for statistical analysis. Data represent means ± SEM. $^{*}P < 0.05$, $^{**}P < 0.01$ versus sham group; $^{\dagger}P < 0.05$, $^{\dagger\dagger}P < 0.01$ versus CLP+vehicle group. CLP, cecal ligation and puncture; Mel, melatonin.
