# Supplementary material for: Melatonin Attenuates Sepsis-Induced Small-Intestine Injury by Upregulating SIRT3-Mediated Oxidative-Stress Inhibition, Mitochondrial Protection, and Autophagy Induction
Source: Front Immunol. 2021 Mar 12;12:625627. doi: 10.3389/fimmu.2021.625627 (PMC8006917; doi:10.3389/fimmu.2021.625627)
Supplement: Supplementary file 4 [file DataSheet_4.pdf]

SUPPORTING INFORMATION

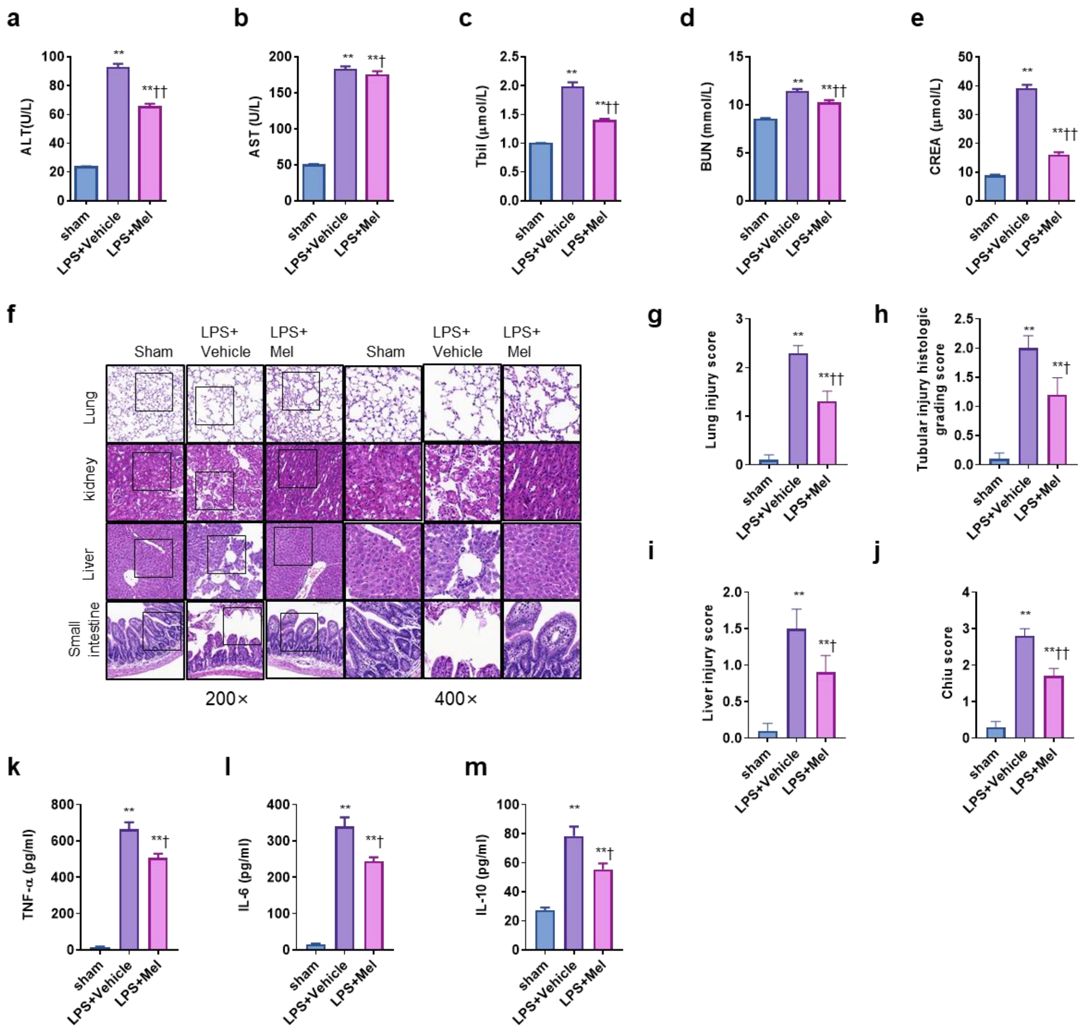

SFigure 3. Melatonin administration enhances multiple-organ function, improves histopathological-injury score, and attenuates inflammatory response in LPS-treated mice. (A-E) Serum indexes of liver function and kidney function; assessed at 8 h after LPS administration. N = 6. A: alanine aminotransferase (ALT); B: aspartate aminotransferase (AST); C: total bilirubin (Tbil); D: blood urea nitrogen (BUN); E: creatinine (CREA). (F) Representative images of lung, kidney, liver, and small intestine after hematoxylin and eosin staining; left and right panels: 200× and 400× magnification images of pathological sections, respectively. (G-J) Quantitative scoring standards of multiple organs: G: lung; H: kidney; I: liver; J: small intestine. The standards for histopathological scores and the degree of multiple-organ injury were evaluated under a microscope by two senior technicians blinded to the treatment protocol. In each tissue sample, 10 random fields

were scored, and the mean value was calculated for statistical analysis. (K-M) Determination of serum inflammatory cytokines: K: TNF-α; L: IL-6; M: IL-10. N = 6. Data represent means ± SEM. $^{**}P < 0.01$ versus sham group; $^{\dagger}P < 0.05$, $^{\dagger\dagger}P < 0.01$ versus LPS+vehicle group. CLP, cecal ligation and puncture; Mel, melatonin; IL, interleukin; LPS, lipopolysaccharide.
