# Supplementary material for: Melatonin Attenuates Sepsis-Induced Small-Intestine Injury by Upregulating SIRT3-Mediated Oxidative-Stress Inhibition, Mitochondrial Protection, and Autophagy Induction
Source: Front Immunol. 2021 Mar 12;12:625627. doi: 10.3389/fimmu.2021.625627 (PMC8006917; doi:10.3389/fimmu.2021.625627)
Supplement: Supplementary file 5 [file DataSheet_5.pdf]

SUPPORTING INFORMATION

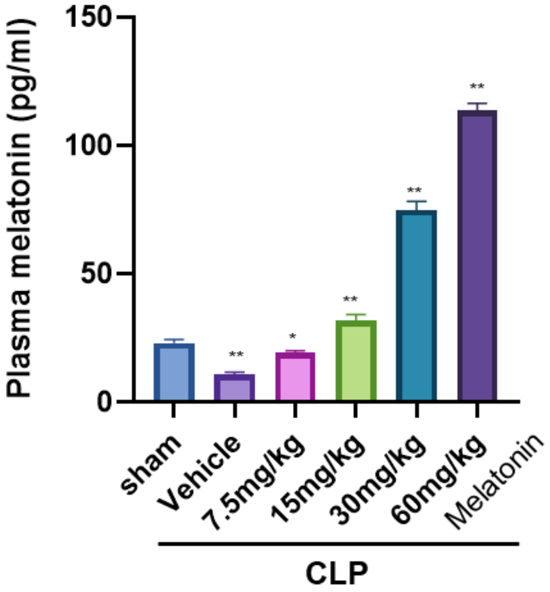

SFigure 4. Plasma melatonin measurement after administration of different doses of melatonin. Plasma melatonin levels increase with increasing melatonin dosage. N = 6. $^{*}P < 0.05$, $^{**}P < 0.01$ versus sham group. CLP, cecal ligation and puncture.
