# Supplementary material for: Melatonin Attenuates Sepsis-Induced Small-Intestine Injury by Upregulating SIRT3-Mediated Oxidative-Stress Inhibition, Mitochondrial Protection, and Autophagy Induction
Source: Front Immunol. 2021 Mar 12;12:625627. doi: 10.3389/fimmu.2021.625627 (PMC8006917; doi:10.3389/fimmu.2021.625627)
Supplement: Supplementary file 6 [file DataSheet_6.pdf]

SUPPORTING INFORMATION

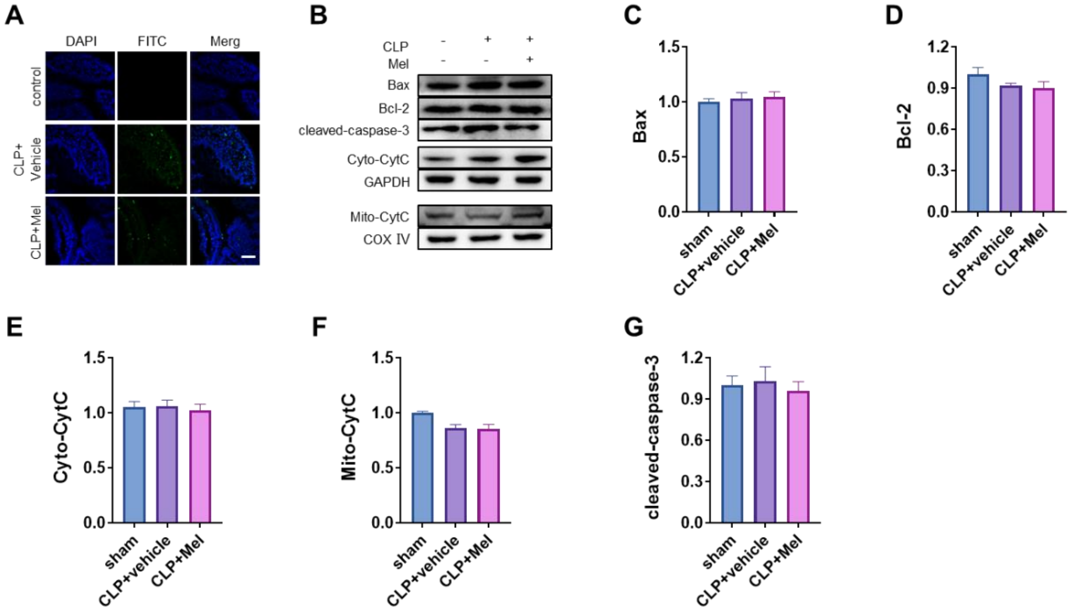

SFigure 5. TUNEL staining and apoptosis-related proteins in small-intestine tissue after CLP treatment. (A) TUNEL staining. Original magnification: 400×. N = 6. (B-G) Representative western blots (B) plus results of densitometric analyses of apoptosis-related proteins Bax (C), Bcl-2 (D), cleaved-caspase-3 (G), and cytochrome C in mitochondria (Mito-CytC; F) and cytoplasm (Cyto-CytC; E). N = 3. Data represent means ± SEM. CLP, cecal ligation and puncture; Mel, melatonin; DAPI, 4′,6-diamidino-2-phenylindole; FITC, fluorescein isothiocyanate; Merg, Merge; TUNEL, terminal deoxynucleotidyl transferase dUTP nick-end labeling; GAPDH, glyceraldehyde 3-phosphate dehydrogenase; Cyto, cytoplasm; Mito, mitochondria; CytC, cytochrome C; COX, cyclooxygenase.
